# Phenotypic and Physiological Responses of Rice Seedlings to Co-Exposure of Polystyrene Microplastics and Heavy Metals

**DOI:** 10.3390/nano16171050

**Published:** 2026-08-23

**Authors:** Ziwen Hao, Xiaolu Bie, Pu Niu, Lin Wang

**Affiliations:** 1 Miami College, Henan University, Kaifeng 475004, China; hzww@henu.edu.cn (Z.H.); 17637720316@163.com (X.B.); 2 School of Marxism, Henan University, Kaifeng 475004, China; 3 College of Geographical Sciences, Faculty of Geographical Science and Engineering, Henan University, Zhengzhou 450046, China

**Keywords:** polystyrene microplastics, heavy metals, cadmium, lead, rice seedlings, element-specific responses, hydroponic experiment

## Abstract

The co-occurrence of microplastics (MPs) and heavy metals in agricultural ecosystems poses emerging threats, yet their combined ecotoxicological effects on crop plants remain poorly understood. To address this gap, a hydroponic exposure experiment was conducted to evaluate the individual and combined effects of 50 mg·L^−1^ polystyrene (PS) microplastics, lead (Pb, 35 mg·L^−1^), and cadmium (Cd, 20 mg·L^−1^) on the phenotypic growth, biomass accumulation, and peroxidase (POD) activity of rice seedlings. The results indicated that: (1) Individual polystyrene microplastics (PS-MPs) treatment did not induce morphological inhibition; rather, it exhibited a growth-promoting trend, with a significant increase in fresh weight and a non-significant increasing trend in dry weight compared to the control. (2) The phenotypic impact of PS-MPs on the toxicity of heavy metals was element-specific. In the PS + Cd co-exposure system, microplastics significantly alleviated Cd-induced inhibition of fresh weight relative to the single Cd treatment, although this recovery effect was not observed in dry weight. Conversely, in the PS + Pb system, microplastics aggravated the phenotypic toxicity of Pb, with biomass showing a numerical decrease relative to the single Pb treatment, though the difference did not reach statistical significance. (3) The plant antioxidant system exhibited organ-specific responses to the combined stresses. Under PS + Pb co-exposure, root POD activity was significantly up-regulated while shoot POD activity was notably suppressed, revealing an asynchrony in physiological responses between roots and shoots. In contrast, the decrease in root POD activity under the PS + Cd system was consistent with phenotypic recovery in fresh weight. In conclusion, PS-MPs can significantly alter the phenotypic and physiological responses of rice seedlings to heavy metals, with the direction of modulation being element-specific. While the underlying mechanisms require further elucidation, this study provides a phenotypic and physiological basis for assessing the early ecological risks associated with the co-exposure of microplastics and heavy metals.

## 1. Introduction

Microplastics (MPs) and heavy metals have emerged as ubiquitous and persistent pollutants in global agricultural ecosystems [1]. Polystyrene (PS), representing one of the major polymer types of MPs, predominantly originates from the degradation of packaging materials and agricultural films, persisting in soil and aquatic media for extended periods owing to its high chemical stability [2]. Concurrently, typical highly toxic heavy metals, such as lead (Pb) and cadmium (Cd), are continuously introduced into farmland systems via sewage irrigation, atmospheric deposition, and the application of agrochemicals [3]. In complex agricultural environments, polystyrene microplastics (PS-MPs) and heavy metals frequently co-occur; consequently, their interactions and combined ecotoxicological effects have become a critical subject in current environmental risk assessments.

Owing to their small particle size, high specific surface area, and surface hydrophobicity, MPs can act as “vectors” for environmental pollutants, binding with heavy metals through physical adsorption, electrostatic interactions, or surface complexation [4]. However, there is currently no consensus regarding the ecotoxicological outcomes of MP-heavy metal co-exposure. Some studies suggest that the adsorption by MPs may enhance the environmental accumulation and bioavailability of heavy metals, thereby leading to toxicity [5]. Conversely, other research indicates that the surface sites of MPs can immobilize free heavy metal ions, reducing their effective concentration in the medium, which manifests as toxicity-mitigating effects [6]. Such directional divergence in toxicity outcomes relies not only on the physicochemical properties of the MPs but is also highly dependent on the specific chemical properties and migration behaviors of the co-existing heavy metals. Therefore, elucidating the phenotypic and physiological response characteristics of plants to the co-exposure of PS-MPs and different heavy metals is pivotal for evaluating the interactive toxicity of such combined pollution.

Rice (*Oryza sativa* L.), a main food crop, is particularly susceptible to becoming a sink for the accumulation of MPs and heavy metals due to its typical flooded cultivation environments (e.g., paddy soils and irrigation water) [7]. The seedling stage represents the most sensitive phase to environmental stress in the rice life cycle. Existing studies have demonstrated that Pb and Cd stress can induce the overproduction of reactive oxygen species (ROS) in rice seedlings, trigger oxidative stress, and impair the plant’s antioxidant defense system by inhibiting the activity of key enzymes such as peroxidase (POD), ultimately suppressing biomass accumulation [8,9,10]. Nevertheless, systematic comparative studies focusing on the differential phenotypic and physiological responses of rice seedlings to the co-exposure of PS-MPs and distinct heavy metals remain scarce.

Therefore, this study utilized rice seedlings as the test organism to establish a hydroponic co-exposure system involving PS-MPs and typical heavy metals (Pb, Cd). By evaluating phenotypic growth indicators (root length, shoot length, and biomass) alongside the activities of key antioxidant enzymes (POD) in both roots and shoots, this study aims to elucidate: (1) the differential regulatory roles of PS-MPs on the toxic effects of Pb and Cd; and (2) the correlation between phenotypic development and physiological responses in rice seedlings under combined exposure scenarios. The findings of this study will provide vital empirical support for a comprehensive understanding of the combined phytotoxicity of MPs and diverse heavy metals, thereby offering a scientific basis for assessing the joint exposure risks of these pollutants in agricultural ecosystems.

## 2. Materials and Methods

### 2.1. Experimental Materials and Setup

Rice seeds (cultivar Guangxingyou 1380) were procured from Hainan Shennong Gene Technology Co., Ltd. (Haikou, China). PS-MPs with a particle size of 50 μm were purchased from Kexinda Polymer Materials Co., Ltd. (Dongguan, China). The PS-MPs were used according to the manufacturer’s specifications as pristine spherical particles without surface functionalization or additives. Although we did not perform independent characterization (e.g., scanning electron microscopy (SEM), Fourier transform infrared spectroscopy (FTIR), zeta potential), the primary objective of this study was to compare the relative phenotypic and physiological responses among treatments rather than to establish a mechanistic link based on particle surface properties. Therefore, the absence of detailed particle characterization does not affect the validity of our comparative conclusions. Heavy metal salts, Cd(NO_3_)_2_·4H_2_O and Pb(NO_3_)_2_, were of analytical grade.

The hydroponic exposure experiments were conducted in 500 mL Erlenmeyer flasks. Each flask contained 250 mL of the respective nutrient or exposure solution and was planted with three morphologically uniform, pre-germinated rice seedlings.

### 2.2. Experimental Design and Culture Conditions

A total of six treatment groups were established, with each group comprising five biological replicates. Control (CK) group: Blank control (Hoagland nutrient solution only), PS group: 50 mg·L^−1^ PS microplastics, Cd group: 20 mg·L^−1^ Cd^2+^, Pb group: 35 mg·L^−1^ Pb^2+^, PS + Cd group: 50 mg·L^−1^ PS + 20 mg·L^−1^ Cd^2+^, PS + Pb group: 50 mg·L^−1^ PS + 35 mg·L^−1^ Pb^2+^. The exposure concentrations were selected based on previous hydroponic studies and preliminary range-finding tests to ensure measurable phenotypic and physiological responses within the 7-day exposure period [11]. Although these concentrations exceed typical agricultural water levels, they are within the range commonly employed in controlled short-term hydroponic toxicity assays for eliciting clear stress responses. The PS-MP suspension was prepared by dispersing PS-MP powder in basal nutrient solution, followed by water-bath sonication (400 W, 20.5 kHz) for 30 min to achieve homogeneous dispersion and reduce particle aggregation.

During the 7-day exposure period, all nutrient solutions were kept static and not renewed. Distilled water was supplemented daily to compensate for evaporative water loss. The initial pH of the Hoagland solution was adjusted to 5.8 ± 0.1. At the end of exposure, the final pH across all treatments ranged from 6.2 to 6.5, and no significant differences in final pH were observed between groups. Continuous pH monitoring or active pH regulation was not performed throughout the experiment after the initial adjustment. Although quantitative measurement of PS-MP sedimentation, aggregation and suspension stability was not conducted, no visible microplastic sediment was observed at the bottom of experimental containers over the 7-day cultivation period.

All experimental flasks were incubated in an environmental growth chamber (BRS-WHM-2000G, Ningbo Plant, Ningbo, China). The controlled culture conditions were set to a constant temperature of 25 °C with a photoperiod of 8 h/day. Throughout the 7-day exposure period, evaporative losses were compensated daily at noon by adding distilled water via a pipette to maintain a constant liquid volume in the flasks.

### 2.3. Preparation of Culture Media and Exposure Solutions

Basal nutrient solution: A modified Hoagland nutrient solution was utilized, consisting of Ca(NO_3_)_2_·4H_2_O (1.18 g·L^−1^), KNO_3_ (0.51 g·L^−1^), MgSO_4_·7H_2_O (0.49 g·L^−1^), and KH_2_PO_4_ (0.14 g·L^−1^), supplemented with trace elements and an iron salt solution (Fe-EDTA) [11].

Microplastic suspension: To prepare the MP suspension, 50.0 mg of PS powder was dispersed in the basal nutrient solution and made up to a final volume of 1 L. To ensure uniform dispersion and prevent particle aggregation, the mixture was subjected to water-bath sonication for 30 min using an ultrasonic disruptor (400 W, 20.5 kHz) to form a stable suspension.

Co-exposure solutions: For the combined pollution treatments, precisely weighed solid Cd(NO_3_)_2_·4H_2_O or Pb(NO_3_)_2_ was directly added to the aforementioned stable PS suspensions according to the designed ratios. The mixtures were stirred thoroughly until complete dissolution prior to reaching the final volume.

### 2.4. Measurement Parameters and Methods

#### 2.4.1. Growth Parameters

Upon completion of the 7-day exposure, the rice seedlings were harvested. The roots were carefully rinsed with deionized water and blotted dry with filter paper to remove surface moisture. Root and shoot lengths were measured using a standard ruler. The fresh weight of the seedlings was recorded immediately, after which the plant tissues were oven-dried at 80 °C to a constant weight to determine the dry weight.

#### 2.4.2. POD Enzyme Activity Assay

The POD levels in the samples were quantified utilizing a specific commercial kit provided by Solarbio Science & Technology Co., Ltd. (Beijing, China). Briefly, fresh plant biomass (roughly 0.10 g) was ground in 1 mL of ice-cold extraction buffer. To obtain the crude enzyme extract, this homogenate underwent centrifugation (8000× *g*, 10 min, 4 °C), after which the clear supernatant was harvested.

Following the supplier’s guidelines, the reaction components were assembled sequentially. The optical density of the mixture was then monitored using a UV-Vis spectrophotometer (721G, Shanghai Yidian, Shanghai, China) at a wavelength of 470 nm. Specifically, spectrophotometric readings were captured exactly at 30 s (A1) and 90 s (A2) to calculate the enzymatic rate [11]. POD activity (U/g) was calculated using the following equation:

POD activity (U/g) = 7133 × (A2 − A1) ÷ W

where W (g) represents the fresh weight of the sample. POD activity is expressed as units per gram of fresh weight (U/g).

### 2.5. Data Analysis

Following preliminary data compilation in Microsoft Excel, variance among the treatment groups was evaluated with SPSS 27.0. Specifically, we applied a one-way ANOVA followed by Duncan’s multiple range test for post-hoc comparisons to determine the significant differences (*p* < 0.05) among treatment groups. Each flask was treated as one independent experimental unit; the three seedlings within each flask were averaged to provide a single value per flask, and five such flasks (n = 5) constituted the replicates for each treatment. All data are presented as mean ± standard deviation (SD). All corresponding data visualizations and figures were plotted using the Origin 2024 platform.

## 3. Results and Discussion

### 3.1. Effects of PS-MPs and Heavy Metals Co-Exposure on the Phenotypic Growth of Rice Seedlings

**Figure 1:**
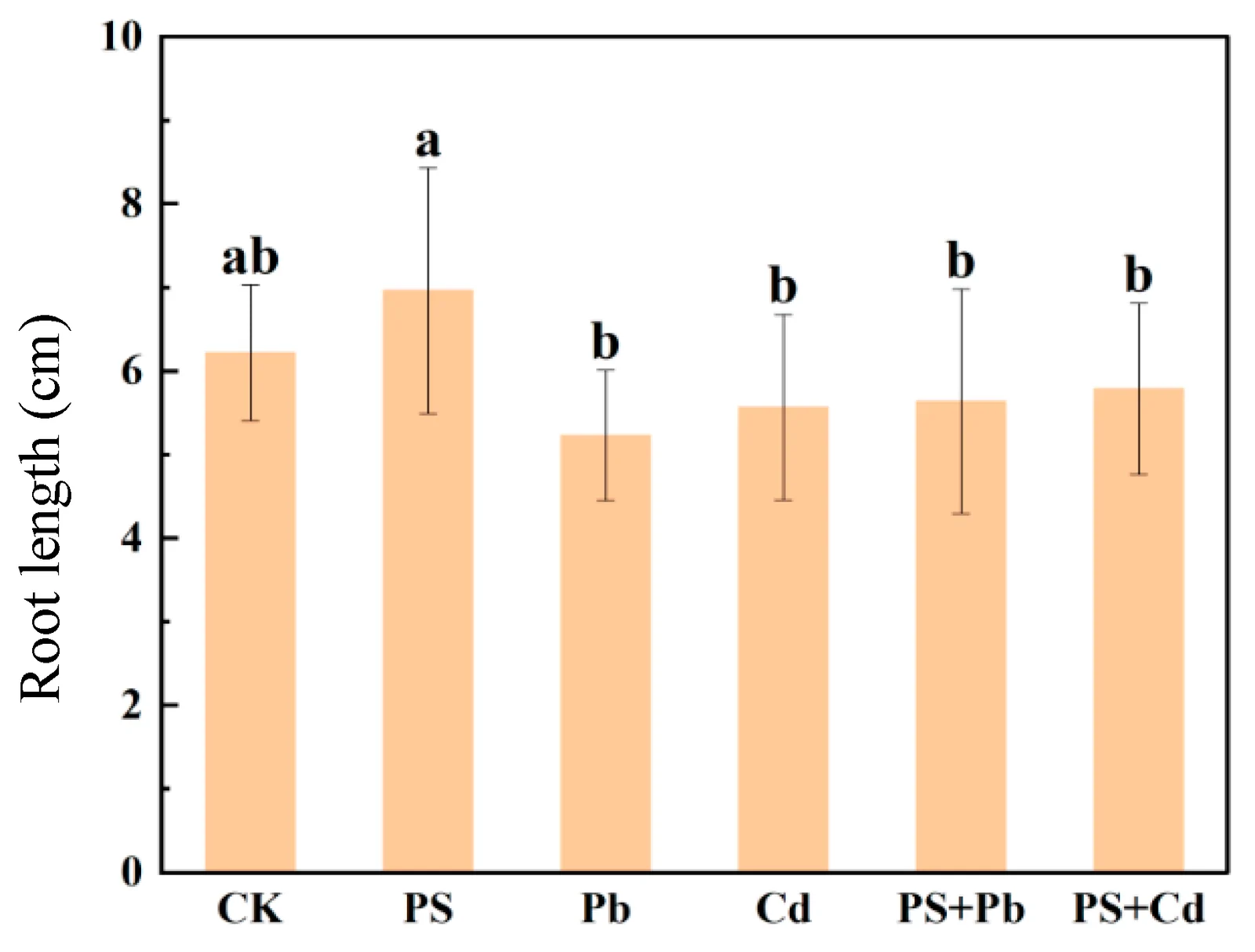
Root length of rice seedlings in response to individual and co-exposure of PS-MPs and heavy metals. Data are presented as mean ± SD (n = 5). Different letters indicate significant differences at *p* < 0.05.

Root and shoot lengths serve as fundamental macroscopic indicators that intuitively reflect the severity of external environmental stress and the corresponding phenotypic responses of plants. As illustrated in Figure 1, the root systems of rice seedlings exhibited highly differentiated morphological responses to the various pollutant exposures. Compared to the CK group, the individual PS microplastic treatment did not inhibit root elongation; rather, it exhibited a marginal growth-promoting trend, resulting in the maximum root length observed among all treatment groups. This phenomenon aligns with multiple previous studies, which demonstrated that low concentrations or relatively large particle sizes of polystyrene microplastics do not exert significant inhibitory effects on the early phenotypic development of rice or wheat seedlings [12,13,14]. This can likely be attributed to the mild physical friction or micro-mechanical stimulation exerted by the microplastic particles on the root epidermis, which subsequently induces compensatory root growth [10,11]. These results indicate that, when present individually, PS microplastics at this specific concentration exert negligible direct morphological toxicity on rice seedlings during early development.

However, upon the introduction of heavy metals, the root elongation of the seedlings was substantially suppressed. As depicted in Figure 1, the root lengths in both the single heavy metal treatments (Pb, Cd) and the co-exposure groups (PS + Pb, PS + Cd) exhibited a downward trend, all of which were significantly lower than that of the individual PS treatment group (*p* < 0.05). Notably, no statistically significant differences in root length were observed between the co-exposure groups and their corresponding single heavy metal treatments. Currently, conclusions regarding the combined toxicity of microplastics and heavy metals remain controversial. Some studies suggest that microplastics can effectively mitigate heavy metal phytotoxicity via surface adsorption [15,16], whereas others argue that microplastics may exacerbate the translocation and toxicity of heavy metals in plants [17,18]. Within the acute hydroponic exposure system employed in this study, neither significant exacerbation nor mitigation by PS-MPs on the morphological toxicity of Pb and Cd was observed. This finding is consistent with certain observations from recent short-term hydroponic exposure studies [19,20].

**Figure 2:**
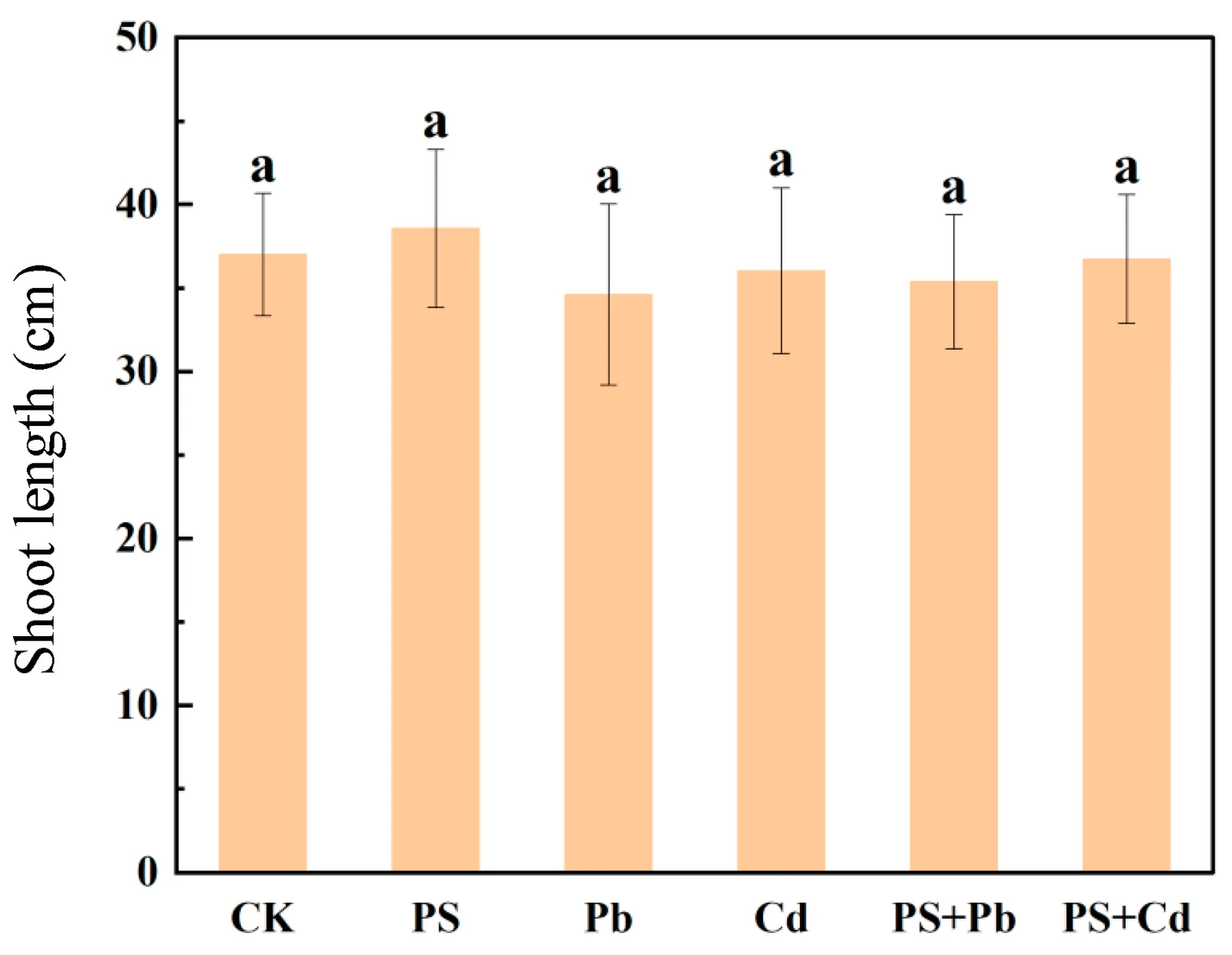
Shoot length of rice seedlings in response to individual and co-exposure of PS-MPs and heavy metals. Data are presented as mean ± SD (n = 5). Letters indicate significant differences at *p* < 0.05.

Regarding shoot development, the results presented in Figure 2 demonstrate that the shoot lengths of the rice seedlings across all treatment groups remained stable, fluctuating between 35 and 40 cm, with no significant differences detected among the groups. This phenomenon indicates that during the 7-day short-term hydroponic exposure, the phytotoxicity induced by the combined pollutants was predominantly confined to the roots, which were in direct contact with the exposure solutions. This result further corroborates previous conclusions regarding the distribution and compartmentalization characteristics of heavy metals and microplastics within plant tissues [17,21]. Serving as the “first line of defense” against external stressors, the plant root system, through its morphological plasticity and the structural interception by the root endodermis, effectively impedes the acropetal translocation and transport of heavy metals and microplastics to the shoots to a certain extent [9]. Consequently, shoot elongation exhibited robust tissue tolerance and a lagging response in the short term, without manifesting apparent damage.

In summary, based solely on apparent morphological elongation indicators (i.e., root and shoot lengths), the introduction of PS microplastics did not significantly alter the macroscopic phenotypic toxicity of heavy metals to rice seedlings. However, the stability of morphological dimensions frequently fails to comprehensively reflect internal disruptions in biomass accumulation and microscopic metabolic impairments within the plant. Whether microplastics might provoke more profound toxicological effects by potentially altering the rhizosphere bioavailability of heavy metals necessitates further in-depth investigation, as the present morphological data alone cannot confirm such vector effects. This should be evaluated in conjunction with biomass accumulation at the biosynthesis level, alongside the response characteristics of the internal antioxidant defense system (e.g., POD activity) at the physiological and biochemical levels.

### 3.2. Effects of PS-MPs and Heavy Metals Co-Exposure on the Biomass of Rice Seedlings

**Figure 3:**
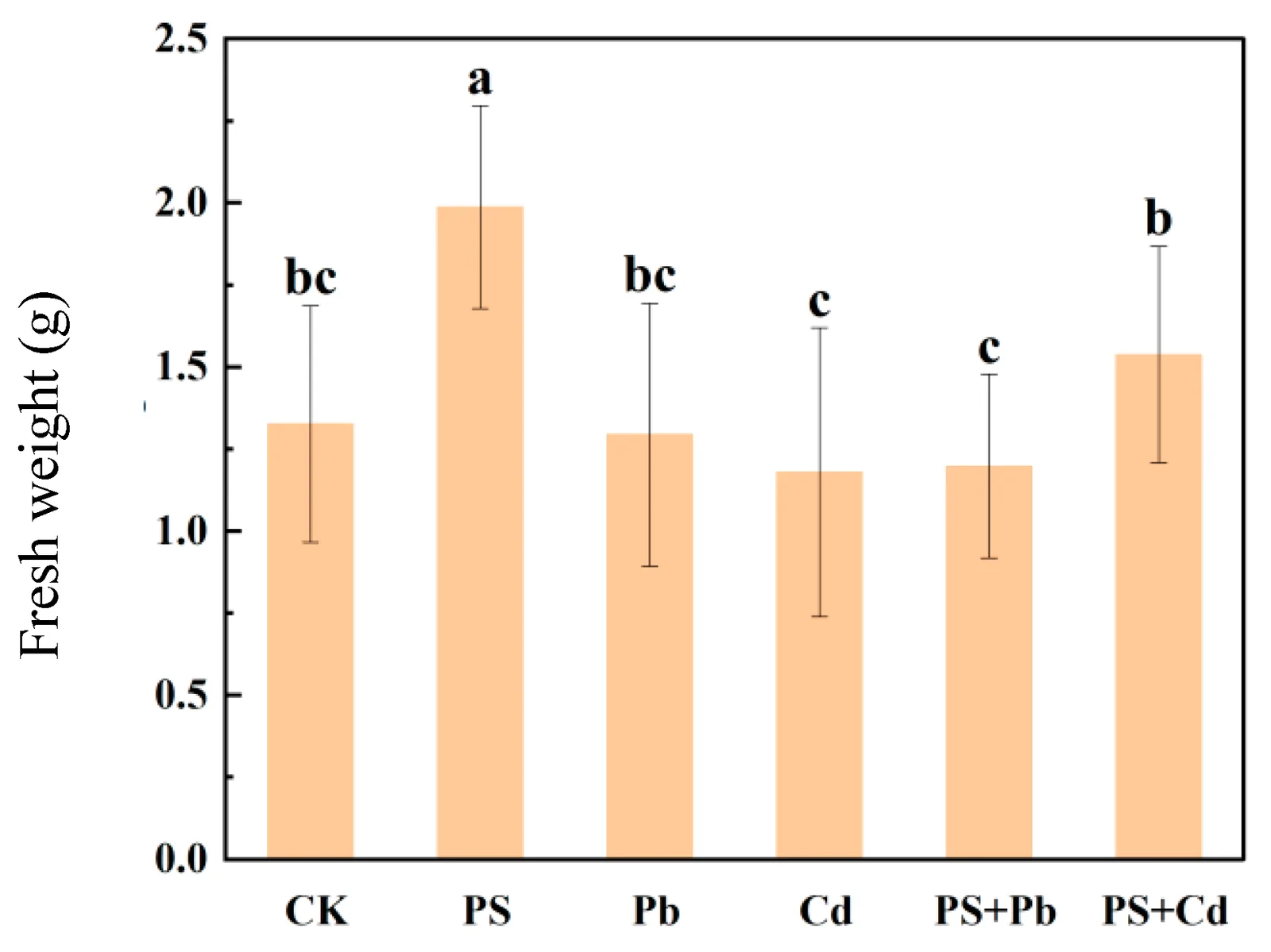
Fresh weight of rice seedlings in response to individual and co-exposure of PS-MPs and heavy metals. Data are presented as mean ± SD (n = 5). Different letters indicate significant differences at *p* < 0.05.

**Figure 4:**
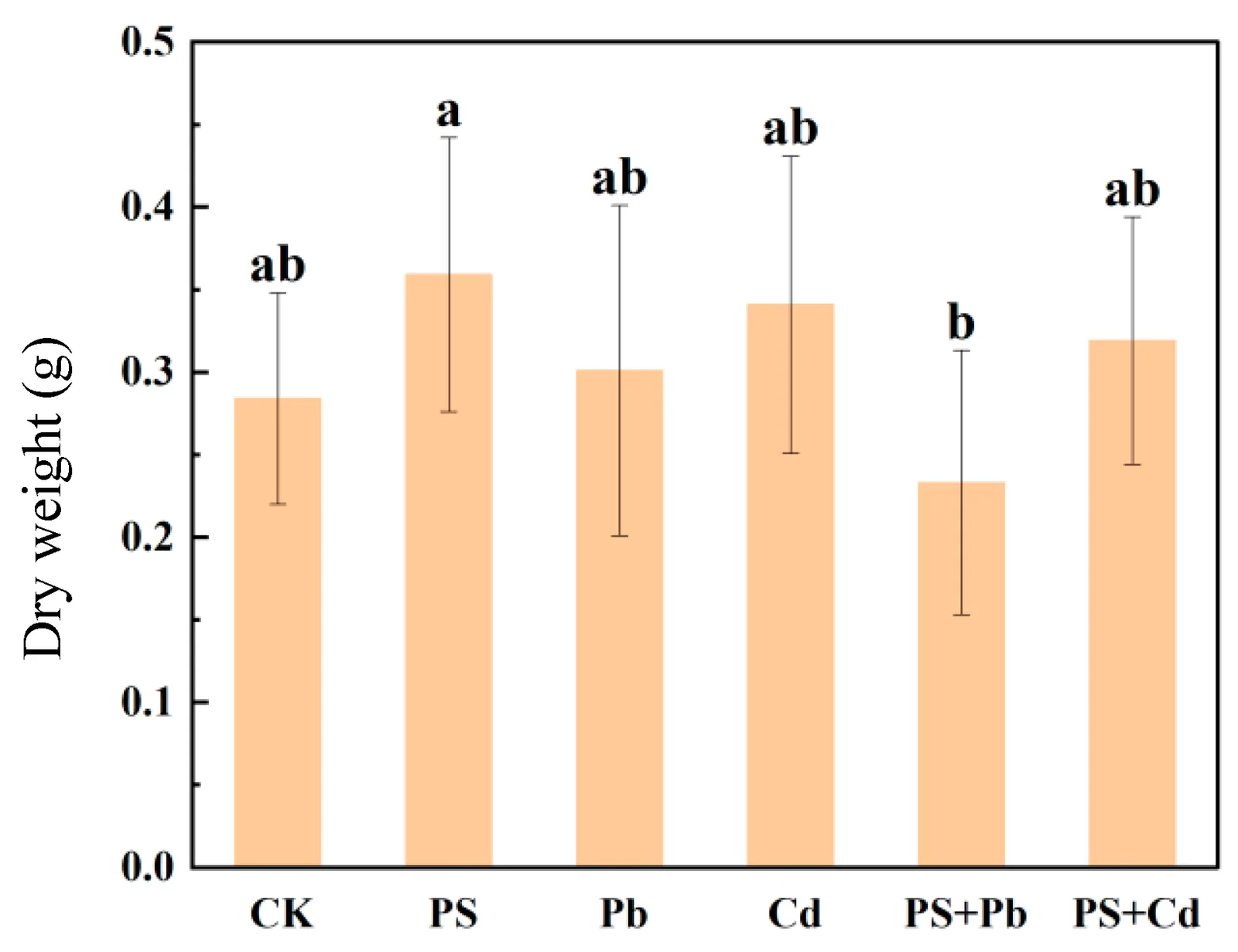
Dry weight of rice seedlings in response to individual and co-exposure of PS-MPs and heavy metals. Data are presented as mean ± SD (n = 5). Different letters indicate significant differences at *p* < 0.05.

Biomass serves as a comprehensive indicator of a plant’s capacity for substance synthesis and accumulation under environmental stress. Compared to one-dimensional phenotypic lengths, biomass more accurately reflects the profound toxicological effects of pollutants on carbon assimilation and fundamental metabolism. As illustrated in Figure 3 and Figure 4, the singular PS-MPs treatment exhibited a pronounced growth-promoting trend in rice seedlings. Specifically, the fresh weight of the PS-treated group was significantly higher than that of the CK group (*p* < 0.05), and its dry weight also reached the maximum value among all treatments (Figure 4). This outcome further corroborates the hypothesis proposed in Section 3.1: under short-term, low-concentration exposure, microplastic particles do not induce direct impairments to biomass synthesis. Similar phenomena have been reported in several recent studies, which suggest that low doses of microplastics can facilitate water and nutrient uptake and accumulation by improving the rhizosphere aeration environment or by providing moderate mechanical stimulation to the root epidermis [22,23,24]. Notably, comparable concentration-dependent stimulatory effects on early seedling growth have also been observed with other types of particulate materials, such as CuO nanoparticles, which promoted germination and seedling development at low concentrations while exhibiting suppression at higher doses [25].

However, in the co-exposure systems, the regulatory direction of PS-MPs on the toxicity of different heavy metals exhibited divergent effects. In the Cd pollution system, microplastics demonstrated a distinct toxicity-mitigating effect. Figure 3 reveals that while singular Cd exposure resulted in a numerical decrease in rice fresh weight relative to CK, this difference was not statistically significant (*p* < 0.05). PS + Pb co-exposure completely counteracted the growth-promoting trend observed under the single PS treatment, resulting in a numerical decrease in biomass relative to the single Pb treatment, though the difference did not reach statistical significance. This alleviation effect is consistent with the observations of Niu et al. [26] and Cong et al. [27]. A plausible speculation is that the PS microplastics may bind with Cd^2+^ in the solution, thereby potentially reducing the concentration of free, highly bioavailable Cd ions in the culture medium, and possibly impeding the transmembrane uptake and toxic influx of Cd by the root system. Nevertheless, direct evidence for reduced Cd^2+^ concentration in the solution and its uptake kinetics is not provided in this study and warrants future investigation.

In contrast, within the Pb pollution system, PS-MPs not only failed to exert any mitigating effects but rather manifested a potential toxicity trend. Data from Figure 3 and Figure 4 indicate that the PS + Pb co-exposure group completely negated the growth-promoting advantage of the pristine PS, with both fresh and dry weights being suppressed to the lowest levels among all groups. Currently, hypotheses regarding the combined toxicity mechanisms of microplastics and heavy metals primarily revolve around the “adsorption-detoxification” and the “Trojan horse” effects [28,29]. In this study, the exacerbated toxicity observed in the Pb system might be tentatively explained by a potential “Trojan horse” effect, although this remains a hypothesis at this stage. Compared to Cd, Pb generally exhibits a stronger surface chemical affinity for microplastics [30]. It is conceivable that Pb-adsorbed microplastic particles attach to the root surface, and the absorbed Pb might undergo localized desorption within the rhizosphere micro-domain under the modulation of root exudates. If this occurs, it could lead to an elevation in the localized effective concentration of Pb at the root surface, which might have exceeded the compensatory regulatory limits of the rice seedlings, thereby contributing to the profound biomass synthesis impairment observed in this treatment. However, direct measurements of Pb speciation or root-surface desorption are required to validate this hypothesis.

Therefore, the results confirm that PS-MPs are not merely inert additives within the combined pollution system; rather, they play a pivotal role as “vectors” that regulate the environmental behavior of heavy metals. By altering the speciation distribution and bioavailability of distinct heavy metals, they mediate contrasting responses. This suggests that variations in apparent biomass are profoundly influenced by the types of external heavy metals and their specific binding-desorption dynamics with microplastics. However, uncovering the internal physiological and biochemical mechanisms driving these biomass disparities necessitates a further micro-level dissection through the response characteristics of the plant’s internal antioxidant defense system (e.g., POD activity).

### 3.3. Effects of PS-MPs and Heavy Metals Co-Exposure on POD Activity in Rice Seedlings

POD is a main antioxidant enzyme in plants responsible for scavenging H_2_O_2_ and mitigating oxidative damage. The variation in POD activity typically exhibits a “biphasic dose–response”: under mild to moderate stress, POD activity is upregulated to scavenge ROS; however, when the stress severity becomes extreme and exceeds the cellular compensatory threshold, the antioxidant system is severely compromised, resulting in a significant decline in enzyme activity or impaired synthesis [31,32].

**Figure 5:**
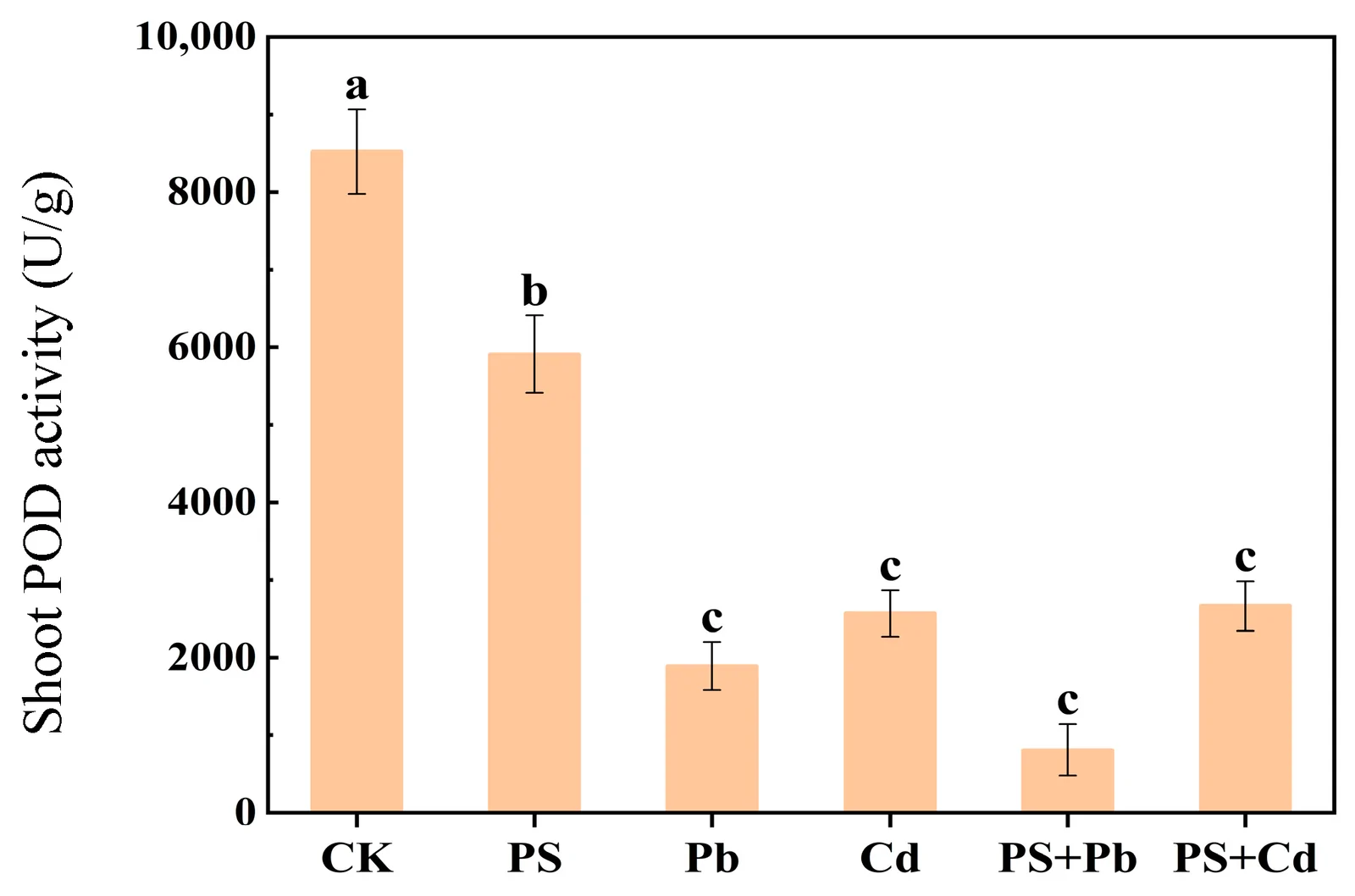
Shoot POD activity of rice seedlings in response to individual and co-exposure of PS-MPs and heavy metals. Data are presented as mean ± SD (n = 5). Different letters indicate significant differences at *p* < 0.05.

**Figure 6:**
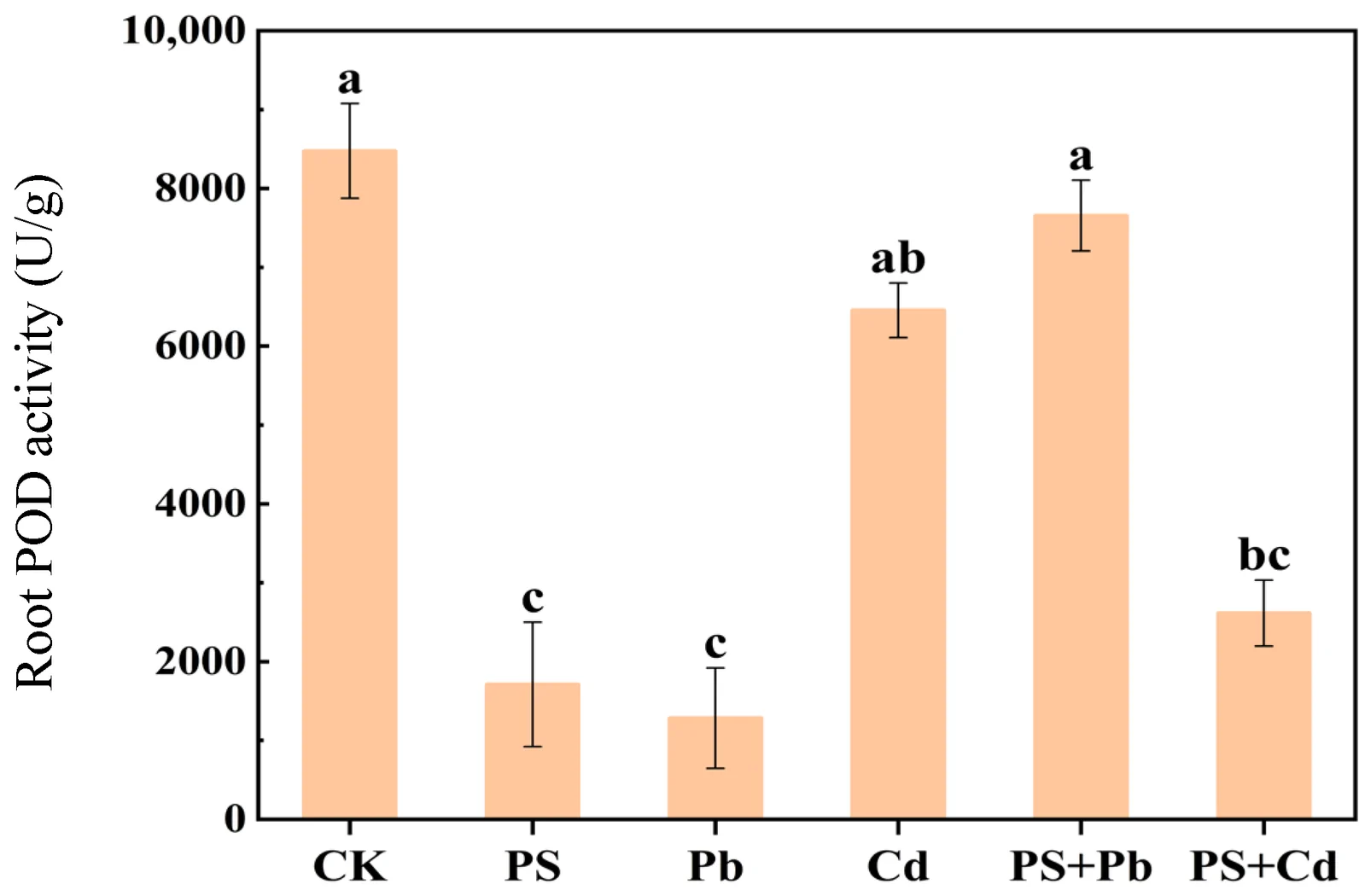
Root POD activity of rice seedlings in response to individual and co-exposure of PS-MPs and heavy metals. Data are presented as mean ± SD (n = 5). Different letters indicate significant differences at *p* < 0.05.

As illustrated in Figure 5 and Figure 6, the POD activities in both the shoots and roots of rice seedlings under the singular PS microplastic treatment were significantly lower than those of the CK group (*p* < 0.05). Combined with the results from Section 3.2, where the singular PS treatment promoted biomass accumulation, this indicates that low-concentration PS-MPs did not trigger an oxidative stress response in the seedlings. Instead, this suggests a growth-promoting trend. This surge is not a sign of toxicity mitigation; rather, it is highly likely a direct biochemical response: when plants are in a highly favorable growth state with low basal ROS levels, they actively downregulate the energy-intensive anabolic metabolism of antioxidant enzymes, reallocating more energy towards biomass accumulation. This is consistent with the findings of Zhang et al. [33], who reported that singular exposure to low doses of conventional polystyrene microplastics generally does not inflict substantial damage on the antioxidant systems of crops like rice.

However, within the co-exposure systems, PS-MPs provoked organ-differentiated and element-specific perturbations in the plant’s antioxidant system induced by heavy metals. In the Pb pollution system, the co-exposure manifested an elevation in localized Pb concentration. Figure 5 reveals that both the singular Pb and PS + Pb treatments led to severe suppression of shoot POD activity, indicating a substantial suppression of the aboveground antioxidant system. Conversely, in the roots (Figure 6), while singular Pb treatment reduced enzyme activity to its lowest, the POD activity in the PS + Pb group exhibited an anomalous and significant surge, rebounding to the highest level comparable to the CK group. This surge might not necessarily signify toxicity mitigation; instead, it could be a biochemical indicator of the root system’s response to a potentially high localized Pb stress. One speculative explanation, as proposed by previous studies [34], is the “Trojan horse”: PS-MPs might concentrate Pb onto the root surface, which could cause the roots to face a sudden heavy metal concentration challenge. This putative acute, localized toxicity might have contributed to the root system’s elevated antioxidant defense response, yet such an elevation alone does not confirm the occurrence of effective mitigation. Nonetheless, this interpretation remains speculative without direct evidence of Pb distribution on the root surface.

In the Cd pollution system, the response of the antioxidant enzymes perfectly corroborated the “adsorption-alleviation” effect of microplastics. Figure 6 demonstrates that under singular Cd exposure, the rice roots maintained high POD activity (with no significant difference from CK), indicating that the roots were actively mobilizing the antioxidant system to combat the oxidative stress induced by free Cd^2+^. However, upon the addition of PS-MPs (PS + Cd group), root POD activity significantly decreased. Aligning with the phenotypic recovery of biomass in this group, this decline in POD activity is consistent with the scenario articulated by Yu et al. [35]: it is plausible that the physical adsorption of heavy metals by microplastics could have reduced the concentration of highly biotoxic free Cd^2+^ in the aqueous phase, cutting off the influx of Cd into the plant at the source, thereby attenuating the trigger for oxidative stress. However, this inference is based on enzymatic data and needs to be corroborated by solution-phase Cd measurements.

Overall, PS-MPs functioned for heavy metal toxicity transmission within the co-exposure systems. By altering the bioavailability of the pollutants, they not only mediated a biochemical toxicity surge in Pb and an alleviation in Cd toxicity but also revealed the asynchrony of plant defense strategies across different spatial dimensions (roots vs. shoots) when coping with combined stresses. This further substantiates that assessing the ecological risks of microplastics cannot be divorced from the specific chemical behaviors of co-existing pollutants.

It should be noted that POD is only one component of the plant antioxidant defense system. Changes in POD activity alone are insufficient to establish the overall level of oxidative stress or the functional status of the entire antioxidant system. Complementary measurements of ROS, H_2_O_2_, malondialdehyde (MDA), superoxide dismutase (SOD), and catalase (CAT) would be required for a comprehensive assessment. Accordingly, the interpretations related to antioxidant responses in the present study are proposed as potential hypotheses rather than definitive conclusions.

## 4. Conclusions

Based on a short-term hydroponic exposure model, this study evaluated the effects of singular and co-exposure of PS-MPs and heavy metals on the phenotypic growth and physiological responses of rice seedlings. The principal conclusions are summarized as follows:

(1) Singular PS-MPs exposure did not induce acute morphological phytotoxicity in rice seedlings; rather, it exhibited a noticeable growth-promoting trend. Fresh weight increased significantly, while dry weight showed a non-significant increasing trend following PS treatment, and POD activity was not significantly upregulated. Collectively, the phenotypic and physiological data indicate that singular microplastic exposure at this dosage did not trigger overt oxidative stress responses.
(2) The phenotypic modulation of heavy metal toxicity by PS microplastics was element-specific. In the PS + Cd system, microplastics alleviated Cd-induced growth inhibition, with a significant recovery in fresh weight relative to the single Cd exposure group (though this recovery was not observed in dry weight). Conversely, in the PS + Pb system, microplastics aggravated biomass suppression relative to the single Pb treatment and abolished the growth-promoting trend observed under singular microplastic treatment.
(3) Co-exposure triggered organ-differentiated responses in POD activity. In the PS + Cd system, a significant decline in root POD activity was observed, which was consistent with the recovery of phenotypic growth in fresh weight. In the PS + Pb system, root POD activity showed an elevation, whereas shoot POD activity was suppressed, highlighting distinct spatial response patterns between roots and shoots.

In conclusion, PS-MPs can differentially alter the early-stage phenotypic and physiological responses of rice seedlings to heavy metals in an element-specific manner. However, this study measured only growth parameters, biomass accumulation, and POD activity, without analyses of metal speciation, adsorption–desorption kinetics, subcellular distribution, or complementary antioxidant and oxidative damage markers. In addition, the PS-MPs were not independently characterized, and the long-term stability of the suspension was not verified. Future studies should incorporate these analyses, along with multi-omics and interfacial chemistry approaches, to provide a more comprehensive assessment of the combined phytotoxicity of microplastics and heavy metals.

## Data Availability

All related data are provided within the manuscript.
